# Postvaccination Multisystem Inflammatory Syndrome in Adult with No Evidence of Prior SARS-CoV-2 Infection

**DOI:** 10.3201/eid2802.211938

**Published:** 2022-02

**Authors:** Young Kyun Choi, Jae Young Moon, Jungok Kim, In Seol Yoo, Geun-Yong Kwon, Heuisoon Bae, Min Seob Song, Sungmin Kym

**Affiliations:** Chungnam National University College of Medicine, Sejong, South Korea (Y.K. Choi, J.Y. Moon, J. Kim, I.S. Yoo, S. Kym);; Sejong Public Health Center, Sejong (G.Y. Kwon);; Sejong City Center for Infectious Diseases Control and Prevention, Sejong (H. Bae);; Inje University College of Medicine, Busan, South Korea (M.S. Song)

**Keywords:** COVID-19, 2019 novel coronavirus disease, coronavirus disease, severe acute respiratory syndrome coronavirus 2, SARS-CoV-2, viruses, respiratory infections, zoonoses, ChAdOx1 nCoV-19 vaccine, multisystem inflammatory syndrome, MIS-A, vaccine-related adverse events, anti-S receptor-binding protein antibody, atrial fibrillation, myopathy

## Abstract

Ten days after receiving the first dose of coronavirus disease vaccine, a 22-year-old woman in South Korea experienced myocarditis, myopathy, pericarditis, and gastroenteritis; rash subsequently developed. There was no evidence of prior infection with severe acute respiratory syndrome coronavirus 2. The diagnosis was multisystem inflammatory syndrome resulting from coronavirus disease vaccination.

Multisystem inflammatory syndrome (MIS) is a serious complication of severe acute respiratory syndrome coronavirus 2 (SARS-CoV-2) infection that affects multiple body systems (cardiovascular, gastrointestinal, skin). It occurs predominantly in children (MIS-C) (*1*) and only rarely in adults (MIS-A) (*2*). The Brighton Collaboration Network (https://brightoncollaboration.us) includes MIS-C and MIS-A as possible coronavirus disease (COVID-19) vaccination–related adverse events (*3*). Most MIS cases occur in persons previously or concurrently infected with SARS-CoV-2 (*4*–*6*). We report a case of MIS-A that occurred after vaccination of a patient with no evidence of prior SARS-CoV-2 infection.

## The Case 

In April 2021, a previously healthy 22-year-old female healthcare worker visited the emergency department of Chungnam National University Sejong Hospital (Sejong, South Korea) with a 2-day history of fever, myalgia, sore throat, diarrhea, and vomiting and a 1-day history of continuous chest pain. She had received her first dose of the ChAdOx1 COVID-19 vaccine (AstraZeneca, https://www.astrazeneca.com) 10 days earlier and had undergone wisdom tooth extraction 8 days earlier. She had no other notable medical history and had not experienced COVID-19 symptoms in the previous 12 weeks. She tested negative for SARS-CoV-2 by real-time reverse transcription PCR. Antipyretics were ineffective.

At initial examination, the patient appeared acutely ill and had an elevated temperature (37.8°C), tachycardia (122 beats/min), mild pharyngeal injection, muscle tenderness, and limb weakness; she exhibited no signs of dental infection. Laboratory tests revealed increased levels of inflammatory markers (Table). Chest radiographs and computed tomography (CT) images showed no signs of lung infiltration; abdominal CT images showed enterocolitis of the small and large intestines. Chest angiography and CT of the lower legs showed no evidence of pulmonary embolism or deep vein thrombosis.

**Table:** Laboratory results for patient with multisystem inflammatory syndrome after coronavirus disease vaccination but no evidence of prior infection with severe acute respiratory syndrome coronavirus 2, South Korea*

Measure	Reference	Hospital day														
		ED visit	1	2	4	8	15	22	29	36	39	50	60	64	68	73
Leukocyte count, × 10^3^ cells/μL	3.5−10.0	12.2	14.7	11.7	28.4	26.5	31.9	58.9	24.3	17.4	29.1	31.5	14.2	19.0	16.0	13.4
Erythrocyte count, × 10^6^ cells/μL	4.0−5.4	3.8	3.2	3.1	3.9	3.1	2.9	2.9	3.3	4.1	4.3	3.9	3.5	3.8	3.6	3.7
Absolute neutrophil count, × 10^3^/μL	1.9−7.4	11.1	13.0	10.3	27.1	24.8	31.4	54.3	22.42	14.7	26.6	19.2	12.1	15.7	13.4	10.2
Absolute lymphocyte count, × 10^3^ cells/μL	1.0−3.9	0.7	0.5	0.8	0.9	0.8	0.6	2.5	1.4	1.4	2.0	0.8	1.2	2.3	1.9	2.6
Platelet count, × 10^3^ cells/μL	130–400	169	159	157	245	168	367	282	274	269	330	177	288	277	182	186
Hemoglobin, g/dL	12–16	11.2	9.6	9.3	11.9	9.3	8.5	8.4	9.4	11.6	12.0	11.4	10.2	11.1	10.5	11.0
Hematocrit, %	36–46	33.7	27.9	28.4	34.2	27.5	24.6	24.2	28.6	34.5	35.9	33.2	30.2	33.1	31.5	33.4
CRP, mg/dL	0–0.5	30.7			>40	26.4	17.3	5.6	25.3	9.5	15.0	11.8	24.2	1.5	0.3	2.3
Procalcitonin, ng/mL	0–0.065	6.524			7.122	1.385	2.851	0.844	2.505	0.272	0.209	2.297	1.747	0.236	0.087	0.134
ESR, mm/h	0–20					49										
IL-6, pg/mL	0–29.4							862.6		54.6	802.1	98.1			621.1	522.1
Ferritin, ng/mL	12–60			13,241		22,860		13,710	12,455			>10,000			4,918	3,595
Lactic acid, mEq/L	0.4–0.8	1.7	1.1	1.4	1.2	1.2	0.7	1.9	0.9							
Sodium, mmol/L	136–145	130	134	139	137	134	131	128	132	137	134	137	138	138	140	141
Potassium, mmol/L	3.5–5.1	3.7	3.9	4.0	2.6	3.8	4.2	4.4	4.7	3.5	2.9	3.0	3.2	3.3	3.0	3.3
Chloride, mmol/L	98–110	100	108	113	104	101	99	99	102	102	94	98	98	99	99	100
CO_2_, mmol/L	19–24	22	21	17	27	19	26	23	25							
Calcium, mg/dL	8.4–10.2	8.1		7.3	7.4	7.5	8.1	7.8	7.9	8.2	8.0	8.2	8.7	8.7	8.7	9.2
Glucose, mg/dL	70–110	114	99	106	94	78	103	121	93	125	81	107	119	105	97	100
BUN, mg/dL	8–20	19.9	11.9	8.7	10.4	12.9	6.1	19.5	12.1	14.6	6.2	10.0	9.1	9.1	6.7	6.2
Creatinine, mg/dL	0.4–0.8	0.77	0.43	0.38	0.38	0.28	0.25	0.26	0.17	0.21	0.23	0.21	0.25	0.32	0.33	0.33
Total protein, g/dL	6.7–8.3	6.1		4.9	4.3	5.1	5.6	5.8	6.3	6.7	6.5	6.3	6.6	6.9	5.9	6.5
Albumin, g/dL	3.8–5.3	3.6		2.8	2.5	2.8	2.9	2.9	2.6	2.8	2.7	2.8	3.1	3.5	3.4	3.9
Total bilirubin, mg/dL	0.22–1.2	0.89		0.30	0.44	0.77	0.53	0.51	0.71	0.40	0.77	0.43	0.69	0.62	0.72	0.77
ALT, U/L	4–44	9		7	7	10	18	22	45	45	53	51	55	60	37	26
AST, U/L	8–38	22		32	31	34	50	43	36	57	50	33	31	54	45	31
ALP, U/L	42–98	58		44	65	84	98	103	185	110	130	137	115	102	80	83
CK, U/L	43–165	33	38	379			20	19	28			23			10	8
CK-MB, ng/mL	0.6–6.3	1.1	1.7	6.1			0.9	0.9	1.1			1.1			0.9	0.8
Troponin I, pg/mL	2.3–17.5	88.7	94.0	286.0		6.9	4.9	4.1							2.5	2.6
NT-proBNP, pg/mL	0–125			4,736		1,707	797	691	8,065	1,246	1,240	1,441			177	123
D-dimer, ng/mL	0–243	>3,200	>3,200		>3,200		>3,200				2,521	1,408			362	282
FDP, µg/mL	0–5	60.09			50.72		27.10									
Fibrinogen, mg/dL	238–498		578		674										203	326
Anti-thrombin III, %	83–128		56		47											
Protein C activity, %	70–130		58		52											
Protein S activity, %	65–140		27		11											
APTT, s	25.1–39.7	34.6	33.9	33.7	33.0	34.6	29.5	30.6	31.9			23.9				
PT, s	9.4–13.6	19.4	18.1	15.9	17.8	19.1	17.9	18.0	14.7			14.0				
INR	0.80–1.23	1.77	1.65	1.44	1.62	1.74	1.63	1.64	1.33			1.27				
Anti-HPF4 antibody	Neg.			Neg.												
LDH, U/L	200–400	511					1,428	1,472				1,161			1,279	1,417
Rheumatoid factor, IU/mL	0–18			8												
C3, mg/dL	65–135			108												
C4, mg/dL	13–35			19												

*ALP, alkaline phosphatase; ALT, alanine aminotransferase; APTT, activated partial thromboplastin time; AST, aspartate aminotransferase; BUN, blood urea nitrogen; C3, complement 3; C4, complement 4; CK, creatine kinase; CK-MB, creatine kinase-myocardial band; CRP, C-reactive protein; ED; emergency department; ESR, erythrocyte sedimentation rate; FDP, fibrin and fibrinogen-degradation products; IL-6, interleukin 6; INR, international normalized ratio; LDH, lactate dehydrogenase; NT-proBNP, N-terminal–pro B-type natriuretic peptide; PT, prothrombin time.

Approximately 6 hours after arrival, the patient’s blood pressure dropped to 70/45 mm Hg. After she received norepinephrine, her blood pressure normalized, and she was transferred to the intensive care unit, where we diagnosed myocarditis and pericarditis. Additional findings were elevated cardiac enzymes, ST segment elevation on electrocardiogram, and a small pericardial effusion on echocardiogram. Cardiac magnetic resonance imaging and biopsy sampling were not performed because of the patient’s hemodynamic instability. PCR results were negative for adenovirus, metapneumovirus, rhinovirus, bocavirus, parainfluenza virus, respiratory syncytial virus, influenza virus, enterovirus, norovirus, rotavirus, astrovirus, and sapovirus, as were results for other tests for viruses causing viral myocarditis. Test results for C-rheumatoid factors and antineutrophil cytoplasmic, P-antineutrophil cytoplasmic, and antinuclear antibodies were also negative.

On hospital day 4, atrial fibrillation with a rapid ventricular response accompanied by hypotension (80/50 mm Hg) developed. After 2 treatments with cardioversion, the patient’s cardiac rhythm reverted to sinus tachycardia, and her blood pressure normalized.

**Figure 1:**
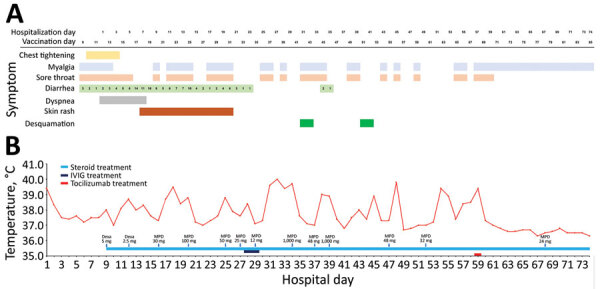
Clinical course of illness in adult with postvaccination multisystem inflammatory syndrome and no evidence of prior SARS-CoV-2 infection, South Korea. A) Signs/symptoms according to the day of hospitalization and the days since vaccination. B) Patient’s maximum body temperature and anti-inflammatory therapy according to the day of hospitalization. Dexa, dexamethasone; IVIG, intravenous immunoglobulin; MPD, methylprednisolone.

On day 7, a generalized macular rash developed and was treated with dexamethasone (5 mg/d for 3 d, followed by 2.5 mg/d for 4 d), after which methylprednisolone was administered for a possible antimicrobial drug–induced eruption. The patient’s fever, rash, and inflammatory marker levels fluctuated according to steroid dose (Figure 1, panel B). Echocardiography images (day 12) showed an increased 1-cm deep pericardial effusion during diastole through the heart circumference without evidence of endocarditis.

**Figure 2:**
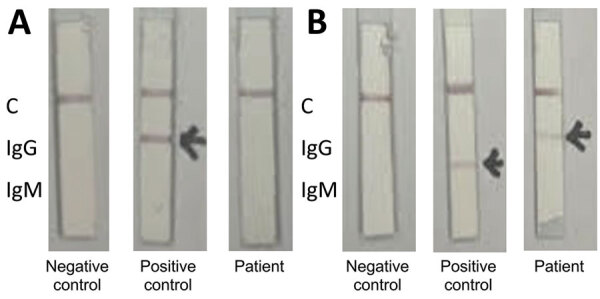
Colloidal gold qualitative immunoassay for antibodies against severe acute respiratory syndrome coronavirus 2, South Korea. A) Nucleocapsid protein conjugate; B) spike receptor-binding domain conjugate. The positive control serum contains antinucleocapsid IgG and anti–spike protein receptor-binding IgM. C, control.

On day 15, SARS-CoV-2 serologic testing with a chemiluminescence immunoassay (Liaison SARS-CoV-2 TrimericS IgG assay; DiaSorin, https://ww.diasorin.com) was performed. Antibody level was 21.88, which is high compared with the average value of 5.56 after first vaccination among healthcare workers without prior SARS-CoV-2 infection but low compared with the average value of 46.34 among those with prior infection (*7*). Antibody analysis using an in-house colloidal gold qualitative immunoassay was positive for anti–spike protein receptor-binding antibodies and negative for antinucleocapsid antibodies (Figure 2).

We empirically administered multiple regimens of antimicrobial drugs during the first 21 days of hospitalization. Bacterial cultures were negative, and no focal signs of infection were found. MIS-A was diagnosed on day 21 after the possibility of infection was excluded, and empiric administration of antimicrobial drugs was discontinued. 

On days 28 and 29, human immunoglobulin therapy (1 g/kg) was administered because after 2 weeks of steroid therapy, the patient’s rash had subsided but her body temperature and C-reactive protein (CRP) level remained high. Muscle weakness, especially hip flexion, had worsened, and the patient was unable to stand without assistance. At that time, the steroid dose was increased, but the disease was not controlled. The immunoglobulin therapy also produced no therapeutic response. The patient’s fever spiked to 40°C, and her CRP level increased. On day 34, steroid pulse therapy (methylprednisolone 1 g/d for 3 d) was initiated, resulting in defervescence and decreased CRP levels. When the steroid dose was tapered, her body temperature and CRP level increased, and steroid pulse therapy was extended for another week. After steroid therapy was discontinued, the patient’s body temperature and CRP levels again increased. She experienced desquamation of the skin on her fingers on day 30 and of her toes on day 40. 

On day 30, a nerve conduction velocity test and electromyogram showed signs of myopathy. Interventional angiography (day 43) showed no abnormality of her coronary arteries. Positron emission tomography (day 59) showed increased contrast medium uptake by soft tissues resulting from inflammation but no focal signs of infection.

On day 60, tocilizumab (8 mg/kg) was administered, after which the patient remained afebrile and the muscle pain in her extremities decreased (Figure 1). She was discharged on day 74 despite residual muscle weakness requiring rehabilitation therapy.

## Conclusions

Most vaccine-related MIS cases have been associated with past or concurrent SARS-CoV-2 infection; recently, MIS cases occurring after mRNA vaccine administration in children and adults in the absence of SARS-CoV-2 infection have also been reported (*8*,*9*). To our knowledge, this case of MIS in an adult was induced by a viral-vector vaccine. This case meets the Brighton Collaboration Criteria for definite MIS-A on the basis of patient age, fever (>3 days), multiorgan involvement, elevated inflammatory markers, elevated N-terminal–pro B-type natriuretic peptide, neutrophilia, lymphopenia, pericardial effusion, and electrocardiographic changes consistent with myopericarditis (*3*).

Antinucleocapsid antibodies typically appear >2 weeks after onset of SARS-CoV-2 infection (*10*), although in some patients they do not appear (*11*). For the patient reported here, at the time she visited the hospital, the cumulative incidence of COVID-19 in her community was 333 cases/100,000 population and the average daily number of cases in the 12 weeks before her visit remained low (n = 1.95). The medical institution where she worked did not treat COVID-19 patients. Given that she had not had COVID-19 signs/symptoms within the previous 12 weeks, the likelihood of prior infection is low.

The clinical features of Kawasaki disease, including desquamation, are similar to those reported for this patient. Desquamation has reportedly occurred in COVID-19 patients, MIS patients, and COVID-19 vaccine recipients (*11*–*13*). Kawasaki disease primarily affects children; gastrointestinal involvement is uncommon, and coronary artery dilatation is the main cardiac problem observed. MIS almost universally involves the gastrointestinal and cardiac systems; incidence of shock and myocarditis/pericarditis is high (*3*). We ruled out adult-onset Still’s disease on the basis of absence of arthritis, liver enzyme levels within reference range, and an inconsistent skin rash (*14*). Features of toxic shock syndrome are also similar to those reported for this patient, including fever, rash, desquamation, hypotension, gastrointestinal symptoms, myalgia, and mucosal inflammation. However, we found no evidence of staphylococcal or streptococcal infection, and the patient had not used tampons. Although we cannot rule out other infections, autoimmune causes, or malignancies, the most reasonable diagnosis for this patient is MIS-A.

MIS mainly occurs after SARS-CoV-2 infection in children. The reason for this age predilection is unknown, but if it is associated with the SARS-CoV-2 spike protein or antibodies induced by the spike protein (the target of SARS-CoV-2 vaccines), vaccine-associated MIS-C may become more common as more children receive SARS-CoV-2 vaccination. 
